# Association of the *IFNG* +874T/A Polymorphism with Symptomatic COVID-19 Susceptibility

**DOI:** 10.3390/v16040650

**Published:** 2024-04-22

**Authors:** Kevin Matheus Lima de Sarges, Flávia Póvoa da Costa, Erika Ferreira dos Santos, Marcos Henrique Damasceno Cantanhede, Rosilene da Silva, Adriana de Oliveira Lameira Veríssimo, Maria de Nazaré do Socorro de Almeida Viana, Fabíola Brasil Barbosa Rodrigues, Mauro de Meira Leite, Maria Karoliny da Silva Torres, Christiane Bentes da Silva, Mioni Thieli Figueiredo Magalhães de Brito, Andréa Luciana Soares da Silva, Daniele Freitas Henriques, Izaura Maria Vieira Cayres Vallinoto, Giselle Maria Rachid Viana, Maria Alice Freitas Queiroz, Antonio Carlos Rosário Vallinoto, Eduardo José Melo dos Santos

**Affiliations:** 1 Laboratory of Genetics of Complex Diseases, Institute of Biological Sciences, Federal University of Pará, Belem 66000-000, Brazil; kevsarges@gmail.com (K.M.L.d.S.); flaviacosta@ufpa.br (F.P.d.C.); erikaferreira.bio@gmail.com (E.F.d.S.); ma_cantanhede@hotmail.com (M.H.D.C.); rosilenesilva2005@yahoo.com.br (R.d.S.); maryvyana@hotmail.com (M.d.N.d.S.d.A.V.); fbrasil.barbosa@yahoo.com.br (F.B.B.R.); mauroleite@gmail.com (M.d.M.L.); chrisbentes@gmail.com (C.B.d.S.); mionibrito@gmail.com (M.T.F.M.d.B.); dealuciana@gmail.com (A.L.S.d.S.); 2 Graduate Program in Biology of Infectious and Parasitic Agents, Federal University of Pará, Belem 66000-000, Brazil; maria.torres@icb.ufpa.br (M.K.d.S.T.); ivallinoto@ufpa.br (I.M.V.C.V.); alicefarma@hotmail.com (M.A.F.Q.); vallinoto@ufpa.br (A.C.R.V.); 3 Center for Biological Health Sciences, State University of Pará, Tucuruí 68455-210, Brazil; adylameira@gmail.com; 4 Laboratory of Virology, Institute of Biological Sciences, Federal University of Pará, Belem 66000-000, Brazil; 5 Graduate Program in Clinical Analysis, Federal University of Pará, Belem 66000-000, Brazil; 6 Section of Arbovirology and Hemorrhagic Fevers, Evandro Chagas Institute, Health Surveillance Secretariat, Brazilian Ministry of Health, Ananindeua 67000-000, Brazil; danielehenriques@iec.gov.br; 7 Malaria Basic Research Laboratory, Parasitology Section, Evandro Chagas Institute, Health Surveillance Secretariat, Brazilian Ministry of Health, Ananindeua 67000-000, Brazil; giselleviana@iec.gov.br

**Keywords:** COVID-19, host genetics, polymorphisms, *IFNG*, TNF

## Abstract

Tumor necrosis factor (TNF) and interferon-gamma (IFNγ) are important inflammatory mediators in the development of cytokine storm syndrome (CSS). Single nucleotide polymorphisms (SNPs) regulate the expression of these cytokines, making host genetics a key factor in the prognosis of COVID-19. In this study, we investigated the associations of the *TNF* -308G/A and *IFNG* +874T/A polymorphisms with COVID-19. We analyzed the frequencies of the two polymorphisms in the control groups (CG: *TNF* -308G/A, *n* = 497; *IFNG* +874T/A, *n* = 397), a group of patients with COVID-19 (CoV, *n* = 222) and among the subgroups of patients with nonsevere (*n* = 150) and severe (*n* = 72) COVID-19. We found no significant difference between the genotypic and allelic frequencies of *TNF* -308G/A in the groups analyzed; however, both the frequencies of the high expression genotype (TT) (CoV: 13.51% vs. CG: 6.30%; *p* = 0.003) and the *T allele (CoV: 33.56% vs. CG: 24. 81%; *p* = 0.001) of the *IFNG* +874T/A polymorphism were higher in the COVID-19 group than in the control group, with no differences between the subgroups of patients with nonsevere and severe COVID-19. The *T allele of *IFNG* +874T/A (rs2430561) is associated with susceptibility to symptomatic COVID-19. These SNPs provided valuables clues about the potential mechanism involved in the susceptibility to developing symptomatic COVID-19.

## 1. Introduction

COVID-19, caused by SARS-CoV-2, is a highly infectious disease, which resulted in 774 million cases worldwide and 7 million deaths from its emergence in December 2019 until January 2023 [1]. Approximately 80% of cases are clinically mild or asymptomatic, while 15% progress to a severe form and 5% to a critical form [2,3], requiring hospitalization and ventilatory support [4,5].

Hospitalized patients have high levels of cytokines and may progress to a predominantly inflammatory form of the disease called cytokine storm syndrome (CSS), a clinical condition characterized by high plasma levels of several cytokines that can trigger systemic inflammatory and thromboembolic processes [6,7,8,9], among which tumor necrosis factor (TNF) and interferon-gamma (IFNγ) stand out, leading to a worse clinical outcome.

TNF is a proinflammatory cytokine produced by different cell types, including alveolar macrophages activated by cell damage caused by SARS-CoV-2, generating a systemic response [10,11]. In this context, IFNγ is a key cytokine in the antiviral response by T helper cells (Th1), stimulating antigens and macrophages to produce proinflammatory cytokines, including TNF [12,13].

Thus, genetic polymorphisms that affect the expression of genes encoding these cytokines (*TNF* and *IFNG*) are natural candidates for prognostic and predictive biomarkers in COVID-19. For this reason, the present study investigated the association of two single nucleotide polymorphisms (SNPs), *TNF* -308G/A (rs1800629) and *IFNG +874T/A* (rs2430561), with aspects of COVID-19 severity, becoming the first study to investigate the association of *IFNG* +874T/A with this infection. These SNPs are loci of quantitative traits and modulators of the expression of these genes [14,15,16], whose relevance in viral diseases has already been demonstrated in the literature [17,18,19].

## 2. Materials and Methods

### 2.1. Study Population, Sample Groups and Sample Collection

We collected data and peripheral blood from 222 patients (COVID-19 group, CoV) diagnosed with COVID-19 clinically and with laboratory tests. The diagnosis was confirmed positive by RT-PCR or antigen test. In addition, positive IgM serology and lung computed tomography suggestive of COVID-19 were present in most patients.

All patients aged 18 years or older, of both sexes, unrelated, residing in the metropolitan area of Belem (State of Pará, Brazil) were recruited between July 2020 and December 2021 from the Arbovirology Department of the Evandro Chagas Institute and in the hospital wing of the Belem Adventist Hospital. Only patients who had not been vaccinated against SARS-CoV-2 at the time of collection were considered eligible. Symptomatic patients were classified into nonsevere and severe COVID-19 subgroups according to the severity criteria of the World Health Organization [4].

Both polymorphisms had been studied in the Belem population in case-control studies prior to the COVID-19 pandemic. Thus, we used the genotypic and allelic frequencies described in the control samples of these studies as representative of the population of Belem (control group, CG) and used them for comparisons with our sample. Three studies were used for the *TNF* -308G/A polymorphism [20,21,22] with a total of 497 individuals, while for the *IFNG* +874T/A polymorphism, we used two studies [22,23] with a total of 397 individuals.

A total of 8 mL of peripheral blood sample was collected using a vacuum collection system containing ethylenediaminetetraacetic acid (EDTA) as anticoagulant. The samples were transported to the Laboratory of Virology of the Federal University of Pará, where the aliquots of whole blood, plasma and leukocytes were separated and stored at −20 °C.

### 2.2. DNA Extraction and Genotyping of the TNF -308G/A and IFNG +874T/A Polymorphisms

Genomic DNA was extracted from 200 μL of peripheral blood using a ReliaPrep™ Blood gDNA Miniprep System kit (Promega, Madison, WI, USA) following the manufacturer’s instructions. The *TNF* -308G/A (rs1800629; 6p21.33) and *IFNG* +874T/A (rs2430561; 12q15) polymorphisms were analyzed by real-time PCR using a StepOnePlus™ Real-Time PCR System. For the *TNF* -308G/A polymorphism, a TaqMan^®^ genotyping assay was (product code: C_7514879_10, Thermo Fisher, Carlsbad, CA, USA) pre-engineered and customized to contain the FAM/VIC probes for the respective alleles. The *IFNG* +874T/A-specific primers (F: 5′-TTC AGA CAT TCA CAA TTG ATT TTA TTC T-3′ and R: 5′-CCC CCA ATG GTA CAG GTT TC-3′) and probes (FAM/VIC: AAAATCAAATC[T/A]CACACACACA-MGB) were previously described [24]. Both reactions used an iTaq Universal Probes Supermix (Bio-Rad, Hercules, CA, USA) and followed a program of 10 min at 95 °C, 40 cycles of 15 s at 95 °C and 1 min at 60 °C. To minimize possible genotyping errors, we randomly chose 20% of the sample to retest for the two markers.

### 2.3. Statistical Analysis

The difference between the mean age of the groups was estimated using the Mann-Whitney test, while the frequency of sex and comorbidities between the groups was compared using Fisher’s exact test. The frequency of symptoms presented during acute COVID-19 was compared between groups using the Wilcoxon paired test. The genotypic and allelic frequencies were estimated by direct counting. The Hardy–Weinberg equilibrium was calculated to assess whether the distribution of observed genotypic frequencies agreed with the expected frequencies. Comparisons of genotypic and allelic frequencies between groups were as follows: control group (CG) versus CoV and nonsevere versus severe group. The genotypic and allelic frequencies of *TNF* -308G/A SNP (GG versus AA + GA, A versus G) and *IFNG* +874T/A (TT versus AA + AT, A versus T) were tested between the study groups by Fisher’s exact test using the odds ratio with a 95% confidence interval (CI) as a measure of association.

Clinical and epidemiological information and genotyping data were stored in a database using Microsoft Excel 2019 software. All tests were performed using GraphPad Prism v.9.3.0 software, and the results were considered statistically significant with a value of *p* < 0.05.

## 3. Results

### 3.1. Epidemiological Characteristics of Individuals

The sample composed of 222 individuals diagnosed with COVID-19 was subdivided into the groups described above and analyzed regarding variables such as sex, age and other clinical data. The clinical, demographic and epidemiological data that characterized the sample are presented in Table 1. 

The age distribution was significantly different between the groups, with older individuals among the critically ill (*p* ≤ 0.0001). Most patients in the noncritically ill group were female, whereas in the critically ill group, the majority were male. However, this difference was not statistically significant (*p* = 0.5663) (Table 1). The prevalence of comorbidities was higher in the groups of critically ill patients (*p* ≤ 0.0001). There was no significant difference in the prevalence of symptoms between the different severity groups in this study (*p* = 0.0739).

### 3.2. Association of SNPs with Symptomatic COVID-19 Susceptibility

No significant association was found between the genotypes and alleles of the *TNF* -308G/A SNP in any of the comparisons made, with the frequencies of the less frequent allele (*A) ranging from 11.87% to 9.91% in the GC and CoV sample groups, while the frequencies of the same allele in nonsevere and severe patients were 10.0% and 9.72, respectively (Table 2).

The *T allele of the *IFNG* +874T/A SNP was less frequent in the CG (24.81%) than among the group consisting of symptomatic COVID-19 patients, whose frequency was 33.56% (Table 2). Importantly, there was no significant difference between the nonsevere and severe patient subgroups. The genotypic frequencies of both SNPs were in Hardy–Weinberg equilibrium in the studied groups.

## 4. Discussion

Our results confirm the previously observed trends that indicate a higher risk for severe COVID-19 in older patients and those with comorbidities [25,26].

Polymorphisms in the *TNF* and *IFNG* genes, particularly those related to the regulation of their expression, are natural candidates to play a role in the pathogenesis and evolution of COVID-19. Although the results did not show an association of the *TNF* -308G/A polymorphism with COVID-19 or its greater severity, we observed that the *T allele of the *IFNG* +874T/A polymorphism is associated with symptomatic COVID-19. However, no effect of this allele on the severity of infection could be detected.

The frequencies obtained from previous studies in the same population (Belem) showed that for the minor allele *A of the SNP rs1800629 (*TNF* -308G/A), the frequency ranged from 8.3% to 14.5% [20,21,22]; given that the frequency for COVID-19 patients in our study was 9.9%, it fits in this range. However, the frequencies of the minor allele *T of the SNP rs2430561 (*IFNG* +874T/A) were 23.7 and 28.4% in previous studies [22,23], which are lower than in our COVID-19 sample (33.5%).

One putative limitation of our study is population substructuring. However, it would affect all genetic loci. While a difference could be observed in *IFNG *+874T/A, the SNP of *TNF -*308G/A displayed very similar allele frequencies across all subsamples. The same was observed in our previous study [27], where only one SNP loci was found to be associated, while the remaining two still displayed similar genotype and allele frequencies.

In a second study approaching long COVID in a different sample of Belem, our group also compared allele and genotype frequencies with those obtained from the literature for Belem [28]. In this study, ten SNPs were used and most of them displayed no differentiation among Belem and patient subsamples.

Therefore, despite the importance of ethnicity in COVID-19, our study provides no evidence of sample substructuring biases introduced by potential ethnic differences among our subsamples.

Previous in silico studies, such as that by Leite et al. [29], showed that the allele frequencies of polymorphisms associated with high cytokine expression were correlated with the daily COVID-19 mortality rate in dozens of countries. Among them were also the alleles with high expression of *TNF* -308 (A) and *IFNG* +874 (T) that correlated positively with COVID-19 mortality. In this study, the Spearman correlation coefficients of both polymorphisms with the mortality rate did not remain significant after a rigorous correction for multiple tests, but interestingly, the correlation coefficient for *TNF* -308G/A was only 0.35, while that for *IFNG* +874T/A reached 0.46. Thus, there is a good agreement between our results and the trend observed in the meta-analysis by Leite et al. [29], because if the *T allele population frequency influences the proportion of symptomatic individuals that may evolve to a worse disease outcome, this allele can indirectly influence the mortality rate.

Additionally, our results differed in part from a previous case-control study conducted in Hong Kong by Chong et al. [19], seeking an association of cytokine gene polymorphisms with the disease caused by SARS-CoV-1 infection in 2003. Among these polymorphisms are the SNPs in the present study. The authors found an association of the *A allele (low-IFNγ-expression allele) of the *IFNG* +874T/A polymorphism with SARS-CoV-1 infection. The frequency of the *A allele among infected individuals was 83.1%, while in the control population, it was 66.3%. A more detailed frequency analysis of the *A allele among Chinese people showed that the frequencies described in several studies were approximately 80% and never reached values as low as 66%.

While the absence of an association between *TNF* -308G/A and COVID-19 outcomes could be attributed to a lack of statistical power due to small sample sizes, it is less likely given the minimal differences in allele and genotype frequencies (less than 2%). Achieving statistical significance would necessitate thousands of patients. Furthermore, the study by Chong et al. [19], conducted on a population with diverse ethnic and demographic backgrounds, supported the lack of association between *TNF* -308G/A polymorphism and susceptibility to COVID-19.

It is known that the response mediated by type II interferons (IFNγ) and other interferons can stimulate ACE2 expression in airway epithelial cells and in enterocytes, promoting the susceptibility of these tissues to SARS-CoV-2 [30,31]. As the *T allele of *IFNG* +874T/A provides a binding site for nuclear factor κB (NF-κB), thus up-regulating IFNγ expression [16], it is possible that this allele significantly contributes and paves the way to the symptomatic manifestation of COVID-19, as its frequency is higher in infected patients than in the general population.

The association with symptomatic COVID-19 and the absence of an association signal for the *T allele of *IFNG* +874 with severity appears to be paradoxical. A lack of statistical power due to a small sample size may play a role. However, it is also plausible to suppose that after the development of symptoms, which is influenced by the *IFNG* +874T/A polymorphism, the disease outcome, in terms of severity, may be more strongly determined by other factors, like genetic ones that are still unknown or environmental and biological factors, like age, comorbidities, sex, nutritional status and access to the healthcare system, which are already highlighted as major factors in the severity of COVID-19 [25,26,32].

The results suggest an association of the *IFNG* +874T/A polymorphism with symptomatic SARS-CoV-2 infection, despite the sample limitation imposed by the absence of a subsample of asymptomatic individuals. The literature suggests that the prevalence of asymptomatic infections can be quite heterogeneous, as demonstrated by a recent comprehensive systematic review [33]. If we consider only the moderate and high-quality studies presented in this meta-analysis, the prevalence of asymptomatic infections in Asian countries such as Japan and China are approximately 60%, while in European countries, the prevalence of asymptomatic infections is often lower at 50%. Coincidentally, the frequency of the *T allele of rs2430561 in European populations is between 35% and 50% and is much lower in Asian countries, such as Japan (7.2%) and China (18%) [29].

Thus, the *T allele of rs2430561 may play a role in the development of symptomatic infection, which, in association with other risk factors such as advanced age and comorbidities, may proportionally increase mortality among carriers of this allele.

## 5. Conclusions

The *T allele of *IFNG* +874T/A (rs2430561) is associated with susceptibility to COVID-19. These SNPs provided valuable clues about the potential mechanism involved in the susceptibility to developing symptomatic COVID-19.

## Figures and Tables

**Table 1:** Characterization of the sample and subsamples regarding demographic, clinical and epidemiological variables. Comparisons were performed between the groups of nonsevere and severe patients.

Variables	CoV *n* = 222 (%)	Nonsevere *n* = 150 (%)	Severe *n* = 72 (%)
**Sex**			
Women	106 (47.75)	74 (49.33) ^a^	32 (44.44) ^a^
Men	116 (52.25)	76 (50.67) ^a^	40 (55.56) ^a^
**Age (years)**			
21–39	77 (34.68)	64 (42.67)	13 (18.06)
40–59	106 (47.75)	70 (46.67)	36 (50.00)
≥60	39 (17.57)	16 (10.66)	23 (31.94)
Mean	46.35	42.92 ^b^	53.50 ^b^
SD	14.36	12.70	15.07
**Comorbidities** ^c^			
Yes	68 (30.63)	34 (22.66) ^d^	34 (47.23) ^d^
No	154 (69.37)	116 (77.34) ^d^	38 (52.77) ^d^
**Ventilatory support**			
No	150 (67.57)	150 (100)	0 (0)
NIV	70 (31.53)	0 (0)	70 (97.22)
IV	2 (0.90)	0 (0)	2 (2.78)
**Symptoms**	% ^e^		
Fever	70.72	69.33	73.61
Cough	69.37	62.00	84.72
Runny nose	38.29	40.67	33.33
Headache	58.11	58.00	58.33
Sore throat	37.39	40.00	31.94
Chest pain	44.14	40.00	52.78
Abdominal pain	21.17	16.67	30.56
Muscle or body pain	58.56	58.67	58.33
Nausea	25.23	19.33	37.50
Vomiting	11.71	10.00	15.28
Diarrhea	43.24	42.67	44.44
Dyspnea	48.65	36.67	73.61
Weakness	54.50	49.33	65.28
Fatigue	61.26	54.67	75.00
Anosmia	48.20	52.67	38.89
Ageusia	46.85	50.67	38.89

CoV = patients with COVID-19; Nonsevere = patients with mild or moderate COVID-19; Severe = patients with severe COVID-19; *n* = number of individuals; SD = standard deviation; NIV = noninvasive ventilation; VI = invasive ventilation; ^a^ Fisher’s exact test, *p* = 0.5663; ^b^ Mann-Whitney test, *p* < 0.0001; ^c^ comorbidities were chronic diseases such as obesity, diabetes, immunodeficiencies and cancer, in addition to chronic cardiovascular, pulmonary, neurological, liver and kidney diseases; ^d^ Fisher’s exact test, *p* = 0.0003; ^e^ Wilcoxon paired test, *p* = 0.0739.

**Table 2:** Genotypic and allelic frequencies of *TNF* -308G/A (rs1800629) and *IFNG* +874T/A (rs2430561). The sample sizes are presented for all groups and subgroups. Genotypic frequencies are presented as absolute values and percentages (in parentheses), while allele frequencies are presented as percentages. All comparisons were performed using Fisher’s exact test.

Locus.	Genotype/Allele	Frequencies (%)				Frequencies (%)			
		GC *n* = 497	CoV *n* = 222	*p* Value	OR (95% CI)	Nonsevere *n* = 150	Severe *n* = 72	*p* Value	OR (CI 95%)
***TNF*** **-308G/A** **(rs1800629)**	**GG**	390 (78.47)	179 (80.63)	0.5518 ^a^	0.8756 (0.5932–1.292) ^a^	121 (80.67)	58 (80.56)	0.9999 ^c^	0.9929 (0.5020–2.072) ^c^
	**GA**	96 (19.32)	42 (18.92)			28 (18.67)	14 (19.44)		
	**AA**	11 (2.21)	1 (0.45)			1 (0.67)	0 (0.0)		
	*A	11.87	9.91	0.3206 ^b^	0.8166 (0.5709–1.171) ^b^	10.00	9.72	0.3206 ^d^	0.8166 (0.5709–1.752) ^d^
***IFNG*** **+874T/A** **(rs2430561)**	**Genotype/Allele**	**GC** ***n* = 397**	**CoV** ***n* = 222**	***p* value**	**OR ** **(CI 95%)**	**Nonsevere** ***n* = 150**	**Severe** ***n* = 72**	***p* value**	**OR ** **(CI 95%)**
	**TT**	25 (6.30)	30 (13.51)	0.0032 ^e^	2.325 (1.315–4.028) ^e^	23 (15.33)	7 (9.72)	0.2991 ^g^	0.5946 (0.2257–1.458) ^g^
	**TA**	147 (37.03)	89 (40.09)			59 (39.33)	30 (41.67)		
	**AA**	225 (56.68)	103 (46.40)			68 (45.33)	35 (48.61)		
	*T	24.81	33.56	0.0012 ^f^	1.531 (1.186–1.968) ^f^	35.00	30.56	0.3910 ^h^	0.8171 (0.5375–1.238) ^h^

CG = control group; CoV = patients with COVID-19; Nonsevere = patients with nonsevere COVID-19; Severe = patients with severe COVID-19; *n* = number of individuals; OR = odds ratio; 95% CI = 95% confidence interval; ^a^ = comparison of GG vs. GA+AA genotype frequencies between CG and CoV group; ^b^ = comparison of G vs. A allele frequencies between CG and CoV group; ^c^ = comparison of GG vs. GA+AA genotype frequencies between Nonsevere and Severe group; ^d^ = comparison of G vs. A allele frequencies between Nonsevere and Severe; ^e^ = comparison of GG vs. GA+AA genotype frequencies between CG and CoV group; ^f^ = comparison of G vs. A allele frequencies between CG and CoV group; ^g^ = comparison of GG vs. GA+AA genotype frequencies between Nonsevere and Severe group; ^h^ = comparison of G vs. A allele frequencies between Nonsevere and Severe group.

## Data Availability

The raw data supporting the conclusions of this article will be made available by the authors without undue reservation.
